# Differential Long-Chain Polyunsaturated Fatty Acids Status and Placental Transport in Adolescent Pregnancies

**DOI:** 10.3390/nu10020220

**Published:** 2018-02-15

**Authors:** Fernanda Carrilho Pinto da Fonseca, Daniela de Barros Mucci, Renata Pereira Assumpção, Henrique Marcondes, Fátima Lúcia de Carvalho Sardinha, Simone Vargas Silva, Marta Citelli, Maria das Graças Tavares do Carmo

**Affiliations:** 1 Instituto de Nutrição Josué de Castro, Centro de Ciências da Saúde, Universidade Federal do Rio de Janeiro, Rio de Janeiro 21941-902, Brazil; nanda_carrilho@yahoo.com.br (F.C.P.d.F.); danimucci@gmail.com (D.d.B.M.); rpaufrj@gmail.com (R.P.A.); marcondes.hm@gmail.com (H.M.); sardinhaflc@nutricao.ufrj.br (F.L.d.C.S.); 2 Instituto de Biologia Roberto Alcantara Gomes, Universidade do Estado do Rio de Janeiro, Rio de Janeiro 20551-030, Brazil; si_vargas@oi.com.br; 3 Instituto de Nutrição, Universidade do Estado do Rio de Janeiro, Rio de Janeiro 20559-900, Brazil; martacitelli@gmail.com

**Keywords:** polyunsaturated fatty acids, placenta, fatty acid transport protein, adolescents, newborns, umbilical cord

## Abstract

Adolescent pregnancy increases risk of adverse perinatal outcomes. Placental delivery of long-chain polyunsaturated fatty acids (LCPUFA) is essential for fetal growth and development. In this pilot study, we aimed to assess maternal and fetal status of fatty acids (FA) measured at birth and the expression of key genes involved in FA uptake, transport and metabolism in the placenta of fifteen adolescents and fifteen adults. FA were quantified by gas-liquid chromatography. Placental expression of FA transporters was assessed by quantitative real-time polymerase chain reaction (qRT-PCR) and peroxisome proliferator-activated receptor gamma (PPARγ) was quantified by Western Blot. Adolescents had lower docosahexaenoic acid (DHA, 22:6 *n*-3) and total *n*-3 FA levels in maternal erythrocytes and placenta, but these were not different in fetal erythrocytes. Arachidonic acid (AA, 20:4 *n*-6) concentration was increased in placenta but lower in fetal circulation. Plasma membrane fatty acid binding protein (FABPpm) and fatty acid transport protein (FATP) 4 mRNA expressions were not different, however FATP1, fatty acid translocase (FAT/CD36) and fatty acid binding protein 3 (FABP3) mRNA and PPARγ protein levels were decreased in placenta of adolescents. Despite significant downregulation of FATP1, CD36 and FABP3, there was only a modest decrease in LCPUFA (10%) and AA (12%) and no difference in DHA content in cord blood, suggesting that FA transfer to the fetus was partially protected by other factors in adolescents from this cohort.

## 1. Introduction

In order to support normal growth and development of critical structures such as the brain and retina, fetuses and newborns require sufficient amounts of *n*-3 and *n*-6 fatty acids (FA), especially the long-chain polyunsaturated fatty acids (LCPUFA) docosahexaenoic acid (DHA, 22:6 *n*-3) and arachidonic acid (AA, 20:4 *n*-6) [1], which must be secured by maternal transfer through the placenta [2]. The mechanisms underlying placental uptake, metabolism and transfer of FA are complex and not fully understood yet, but have been shown to encompass several membrane-bound and cytosolic fatty acid-binding proteins expressed in the trophoblast [2].

In maternal circulation, FA are primarily transported in lipoproteins, which become available after being released from the lipid backbone through the action of triglyceride hydrolases [3]. Non-esterified FA (NEFA) can enter the trophoblast by passive diffusion, however FA uptake is thought to largely rely on proteins bound to the microvillous plasma membrane: FATPs (fatty acid transport proteins), FABPpm (plasma membrane fatty acid binding protein) and FAT/CD36 (fatty acid translocase) [4,5,6]. CD36 and FATPs are also expressed in the basal membrane of the syncytiotrophoblast and are implicated in the transfer of FA from the intracellular compartment to the fetal circulation [2,7]. It has been suggested that the enrichment of LCPUFA in the fetal circulation relative to the maternal side is especially related to the action of placental FABPpm [8] and FATP4 [9]. FATP1 and FATP4 exhibit both FA transport and acyl CoA synthetase activities, facilitating their trafficking and metabolization within the cell [9].

Trophoblasts also express several FA binding protein (FABP) isoforms [10]. These proteins are thought to mediate FA transport within the cytosol, where they can be oxidized or re-esterified and stored, or directed towards the fetal circulation via the placental basal membrane [11]. Even though little is known about the specific roles of FABP isotypes in the human placenta, it has been demonstrated in rodent trophoblast cells that FABP3 regulates the transport of *n*-3 and *n*-6 PUFA [12]. Fatty acid-activated transcription factors are important regulators of lipid metabolism and are also implicated in the control of these fatty acid transport/binding proteins and placental functions [13]. The peroxisome proliferator-activated receptor (PPAR)γ, in particular, is critically essential for placental development and function and has been suggested to increase placental fatty acid uptake [14].

Despite its important impact in offspring health, little is known about placental lipid transport in the context of adolescent pregnancy age. Pregnancy during adolescence is considered a public health issue in Brazil and other countries, mainly because it is associated with increased risk of adverse outcomes for both mother and child [15], including premature delivery and low birth weight [16,17]. This has been attributed in part to poorer maternal nutritional status and gynecological immaturity, especially among very young mothers (<15 years or <2 years post-menarche) [17]. Moreover, recent evidence suggests that placental nutrient transfer may be altered in adolescent pregnancy. In this context, Hayward et al. have demonstrated that the classical amino acid transport System A, a well-characterized biomarker of placental function, is lower in teenagers compared with adults [18,19]. However, research on the expression of placental proteins involved in FA uptake and trafficking in adolescent pregnancies remains unexplored.

In our previous report [20], we have found that adolescents had higher *n*-6 LCPUFA concentration but lower levels of the *n*-3 LCPUFA eicosapentaenoic acid (20:5 *n*-3) in umbilical cord plasma, despite similar circulating levels between adult and adolescent mothers, suggesting that placental transfer of specific fatty acids, implicated in fetal growth and development, could be altered. It has been suggested that erythrocytes may act as reservoirs of LCPUFA and play a major role in their transport to the fetus [21,22], which make them good biomarkers of FA status during pregnancy. Thus, the present study was addressed to analyze the FA profile of maternal and umbilical cord erythrocytes in adolescent pregnancies and investigate the mRNA expression of key membrane-bound and cytosolic FA transporters thought to mediate LCPUFA transfer across the placenta. The placental FA profile and protein content of PPARγ were also analyzed. We hypothesize that altered LCPUFA status and placental transfer are present in adolescent pregnancies and that differential expression of fatty acid transporter genes and PPARγ could be involved.

## 2. Materials and Methods

### 2.1. Study Population

This is a cross-sectional study of a convenience sample of pregnant adolescents and pregnant adults recruited from July 2013 to April 2014. All participants were attending the Maternity School of Universidade Federal do Rio de Janeiro (MSchUFRJ), a public Institution located in Rio de Janeiro, Brazil, that holds a public health program for adolescents (“Programa de Saúde do Adolescente”’, PROSAD). The research protocol was approved by the Institution’s Ethics Committee (Number 00140361000-10) in accordance with the National Health Council resolution 196/96 [23] and signed informed consent was given by each participant or their legal guardian before engaging in the study.

The inclusion criteria for enrollment in the study were: maternal age from 15 to 19 years for adolescents and from 20 to 40 years for adult women; gestational age between 26 and 28 weeks; absence of chronic and/or pregnancy-related diseases; and no use of dietary supplements containing *n*-3 or *n*-6 polyunsaturated fatty acids for at least 6 months. Women were ineligible to participate in the study in case of non-singleton pregnancy, gestational diabetes, pregnancy-induced hypertension, genetic disorders, maternal infection, current use of medication known to interfere with lipid metabolism, drug or alcohol, smoking, complications during delivery or any diagnosed fetal complications.

A total of 66 pregnant women (25 adolescents and 41 adults) attending regular prenatal care at MSchUFRJ during the recruitment period complied with the inclusion criteria. To conduct this pilot study, a subset of women was randomly selected and a total of 15 pregnant adolescents and 15 pregnant adults enrolled in the study. Maternal data on socio-demographic characteristics, age, medical history, weight and height were collected by means of an interview-administrated questionnaire and consulting medical records. Gestational age was calculated based on the last menstrual period and confirmed by ultrasound. Newborn characteristics (sex, weight, length and head circumference) were also recorded at birth.

### 2.2. Sample Collection and Processing

Placenta samples of the 30 participants were promptly collected by well-trained professionals at the time of vaginal delivery (22 samples) or Caesarean section (8 samples). Total placental weight was recorded after removing the umbilical cord and trimming off fetal and maternal membranes. A small piece of approximately 2.0 × 2.0 × 0.5 cm was excised from the middle of the radius of the placenta, washed two times in cold NaCl solution (0.9%, 4 °C) to eliminate blood residues, frozen in liquid nitrogen and then stored at −80 °C until gene expression and lipid analysis.

At the time of delivery, maternal venous blood (5 mL) was collected intravenously and blood from the umbilical cord was obtained by manual expression before expulsion of the placenta. Both samples were collected using tubes containing 1 g Na_2_-EDTA/L. Plasma was separated from erythrocytes by centrifugation at 3000 rpm/10 min, and the packed erythrocytes were washed three times with isotonic NaCl solution, after removal of the buffy coat. The washed erythrocytes were suspended in an equal volume of isotonic NaCl and stored with 1.0% Na_2_S_2_O_4_ at −80 °C to avoid oxidation of unsaturated fatty acids [24] until the analysis.

### 2.3. Fatty Acids Analysis

The lipid extraction, saponification and transesterification was performed according to the Lepage and Roy method [25] using tridecanoic acid (13:0, Sigma, Cream Ridge, NJ, USA) as internal standard. Fatty acid methyl esters were separated and quantified on an Agilent Technologies 7890A CG chromatograph equipped with a hydrogen flame ionization detector (FID) and an SP 2560 capillary column (biscyanopropyl-polysiloxane, 100 m × 0.25 mm × 0.20 μm; Supelco, Bellefonte, PA, USA), using the EZChrom Elite CDS software (Agilent Technologies, Inc., Santa Clara, CA, USA). The chromatographic conditions were similar to those described by de Velasco et al. [26]. The fatty acid methyl esters were identified by comparison of relative retention times with authentic standards (GLC 463 Reference Standard, Nu-Check Prep. Inc., Elysian, MN, USA) and quantified by comparison with the internal standard.

### 2.4. Placental Gene Expression Analysis

Placenta samples were homogenized in Trizol reagent (Invitrogen, Waltham, MA USA) with a tissue grinder and total RNA was isolated and purified following the protocol described by Rio DC et al. [27], using DNAse treatment. The RNA concentration was determined by spectrophotometer measurement at A260 and cDNA was generated from 1 μg RNA using High Capacity cDNA Reverse Transcription Kit (Applied Biosystem, Foster City, CA, USA). qRT-PCR was performed using the Power Sybr Green Master Mix Kit (Life Technologies, Waltham, MA, USA) and triplicates were analyzed in a Step One Plus system (Life Technologies, Waltham, MA, USA) under the following conditions: 50 °C for 2 min, 95 °C for 10 min and 40 cycles of 95 °C for 15 s and 60 °C for 1 min. The relative expression of genes was calculated by comparison to one calibrating sample from the control group, after Ct values were normalized to glyceraldehyde-3-phosphate dehydrogenase (GAPDH). Melt curve analysis was used to confirm single amplicon production for each gene. The specific primers for FATP1, FATP4, FABPpm, FAT/CD36, FABP3 and GAPDH were designed using the Primer Express Software (Applied Biosystems, Foster City, CA, USA) or Primer3 Software and are listed in Table 1.

### 2.5. Western Blot

The total protein content of placenta extracts was determined by Bradford’s method [28]. Ten μg of total protein were resolved in 10% or 12% SDS-PAGE and transferred to polyvinylidene difluoride membranes after electrophoresis. Protein ladders were run in parallel to estimate molecular weights. Membranes were blocked with 5% bovine serum albumin in PBST (Phosphate Buffered Saline, 0.1% Tween 20) and incubated overnight at 4 °C with primary antibodies against PPARγ (1:2000; Abcam, Cambridge, MA, USA), FATP1 (1:1000; Abcam, Cambridge, MA, USA), FATP4 (1:1000; Abcam, Cambridge, MA, USA) and actin (1:1000; Santa Cruz Biotechnology, Santa Cruz, CA, USA). Membranes were washed, incubated for 1 h with the appropriate biotin-conjugated secondary antibody (1:2000; Santa Cruz Biotechnology, Santa Cruz, CA, USA) and then incubated with horseradish peroxidase-conjugated streptavidin (1:1000). Immunoreactive proteins were visualized using the ECL system. Films were scanned and bands were quantified by densitometry with ImageJ 1.34 s Software (NIH, Bethesda, MD, USA), using actin as internal control.

### 2.6. Statistical Analysis

Data are expressed as mean ± standard deviation (SD), mean ± standard error of the mean (SEM) or n (%). Comparisons between adults and adolescents and between maternal and fetal data from the same group were performed by *t* test or Mann-Whitney, depending on the nature of the variables (normal distribution or not, respectively). Differences were considered statistically significant when *p* < 0.05. Statistical analyses were performed with PRISM 5.01 software (GraphPad Software, La Jolla, CA, USA).

## 3. Results

As opposed to adult women, most adolescents had fewer years of education and did not work during pregnancy. Differences between groups regarding age, marital status and parity were also observed. The average number of prenatal follow-up visits was similar between adolescent and adult pregnant women with the majority being considered in the appropriate prenatal care category (≥6 visits) (Table 2). In both groups, the majority of women had adequate pre-pregnancy nutritional status. The average gestational age at delivery was one week shorter (*p* = 0.023) in adolescents compared to adults, although all babies were born at term within the normal range of birthweight (2500 g to 4000 g) and were adequate for gestational age (AGA). In addition, head circumference as well as length at birth were adequate for most newborns and placental weight was similar in both groups (Table 3). 

Placental levels of DHA (*p* = 0.001) and total *n*-3 PUFA (*p* = 0.01) were significantly lower in the adolescent group compared to adults. In contrast, placenta from adolescents had increased contents of total *n*-6 PUFA, AA and its precursor linoleic acid (LA, 18:2 *n*-6), as well as total essential polyunsaturated FA (EFA, *p* < 0.05 for all). Significant increase was also seen in oleic acid (*p* < 0.05), total monounsaturated FA levels (*p* = 0.04) and the total *n*-6/*n*-3 ratio (*p* = 0.0007) in adolescent placentas. In addition, the tissue ratio of DHA to alpha-linolenic acid (ALA, 18:3 *n*-3), an indicator of *n*-3 LCPUFA biosynthesis, was significantly lower in adolescents (*p* = 0.01) (Table 4).

The contents of oleic acid (18:1 *n*-9), EFA (sum of 18:2 *n*-6 and 18:3 *n*-3) and 22:5 *n*-3 were higher in maternal erythrocytes than in umbilical cord of both groups (*p* < 0.001) (Table 5). On the other hand, 18:0, total saturated FA, AA, total LCPUFA levels and the DHA/ALA ratio were significantly increased in umbilical cord compared to maternal erythrocytes, in both groups. Only in adolescents, umbilical cord erythrocytes also showed higher relative amounts of DHA, 14:0 and 16:0, but lower 22:4 *n*-6, total *n*-6 PUFA and *n*-6/*n*-3 ratio. In comparison with adults, adolescents had lower umbilical cord levels of AA; moreover, total LCPUFA concentration and DHA/ALA ratio were decreased both in maternal and in umbilical cord erythrocytes. Erythrocytes of adolescent mothers also showed higher 22:4 *n*-6 and total *n*-6/*n*-3 ratio, but lower DHA and total *n*-3 PUFA contents compared to adults. No significant difference was observed in the conversion ratio of AA/LA (Table 5).

**Figure 1:**
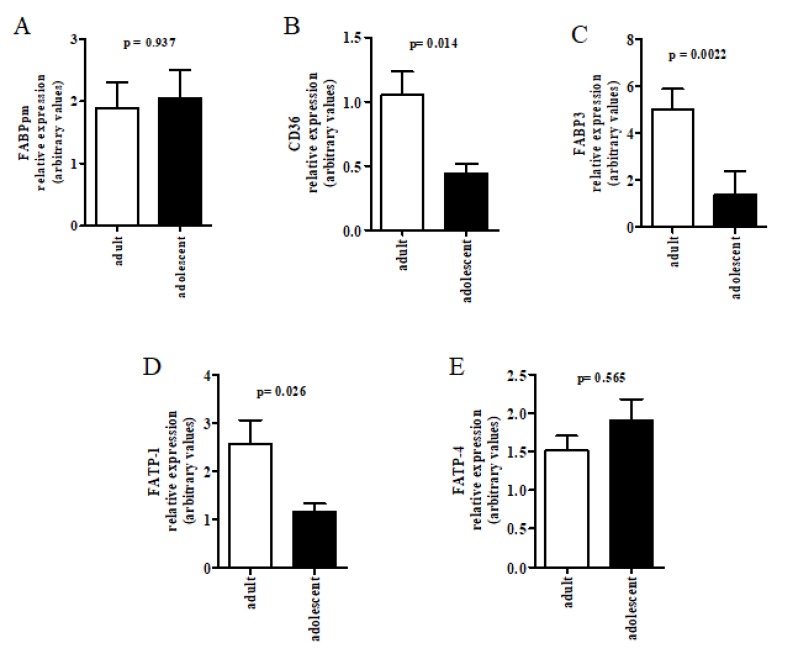
Placental gene expression of (**A**) FABPpm; (**B**) FAT/CD36; (**C**) FABP3; (**D**) FATP1 and (**E**) FATP4 was evaluated in adolescent (*n* = 15) and adult mothers (*n* = 15) after normalization with reference gene GAPDH. Quantification was performed by qRT-PCR. Results are expressed as relative expression mean ± SEM. *p* < 0.05 was considered significantly different according to unpaired *t* test.

**Figure 2:**
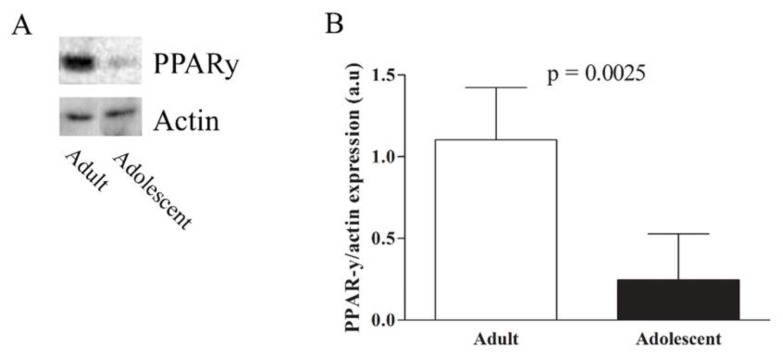
(**A**) Placental protein expression of PPARγ in adults (*n* = 7) and adolescents (*n* = 6); (**B**) Placental densitometric analysis of PPARγ after normalization by actin. Results are expressed as mean ± SD. *p* < 0.05 was considered significantly different according to unpaired *t* test.

Placental gene expression of CD36, FATP1 and FABP3 was significantly lower (respectively *p* = 0.005, *p* = 0.02 and *p* = 0.003) in adolescents compared to adults, whereas FABPpm and FATP4 mRNA was unaltered (Figure 1). The results of FATP1 and FATP4 gene expression were confirmed at protein level, analyzed by Western Blot, showing decreased FATP1 and no difference in FATP4 protein expression in adolescent placentas (Supplementary Figure S1). PPARγ placental protein level was also significantly lower in adolescents than in adults (*p* = 0.002) (Figure 2).

## 4. Discussion

The present study shows interesting findings regarding fatty acids status/transport and pregnancy outcomes in adolescents compared to adults, at delivery: (1) lower DHA and *n*-3 PUFA concentration in maternal erythrocytes and placenta, but no significant difference in umbilical cord levels; (2) AA content was higher in placenta, but lower in fetal erythrocytes; (3) total LCPUFA levels were mildly reduced in maternal and umbilical cord erythrocytes; (4) decreased placental expression of PPARγ protein, FATP1 mRNA and protein, CD36 and FABP3 mRNA levels, but no difference in FABPpm mRNA and FATP4 mRNA and protein expressions; (5) despite having higher rates of insufficient gestational weight gain and slightly decreased gestational age, placental weight and birth outcomes were normal. 

Corroborating our findings, lower levels of DHA, total *n*-3 PUFA and LCPUFA have been previously reported in Brazilian pregnant adolescents of similar socioeconomic condition using erythrocyte FA profile to assess PUFA status [29]. Even though we have not directly accessed the participant’s dietary intake, we speculate that this could be related to the lipid composition of maternal diet, as evidence on the dietary pattern of Brazilian pregnant and lactating adolescents suggest relatively high intake of *n*-6 PUFA, especially LA, and low consumption of *n*-3 LCPUFA, particularly DHA [29,30,31]. In addition, endogenous *n*-3 conversion of ALA to DHA may be inhibited by high dietary levels of the *n*-6 precursor LA, since both ALA and LA compete as substrates for ∆-6 desaturase, and this may further enhance the reliance upon maternal dietary intake of preformed DHA to meet fetal demands [32]. It should be noted that the physiological increase in maternal body fat depots during the first two thirds of gestation allows the accumulation of LCPUFA stores in adipose tissue which become available for placental transfer during late pregnancy, when fetal requirements are higher [33]. This highlights not only the vulnerability of LCPUFA supply to poor maternal habitual intake but also the importance of the placenta in actively concentrating and channeling AA and DHA from the mother to the offspring to ensure the relative enrichment of these LCPUFA in the fetal circulation [33]. In this regard and in agreement with previous studies from our group [20] and others [22], higher values of AA and DHA were detected in cord erythrocytes compared to maternal erythrocytes, in both groups, although statistical difference with respect to DHA was only observed in pregnant adolescents.

In the present study, the DHA/ALA ratio was significantly lower in the placenta and in both maternal and umbilical cord erythrocytes from adolescent pregnancies. Despite the lower *n*-3 bioconversion index in maternal and fetal pools from adolescent pregnancies, no significant difference was detected in umbilical cord DHA concentration. On the other hand, lower AA concentration was detected in fetal erythrocytes from adolescent pregnancies, even though there were no significant differences in AA content from maternal erythrocytes and in bioconversion rates of *n*-6 PUFA, as evidenced by similar AA/LA ratios in all three compartments. These results suggest that LCPUFA biosynthesis capacity had little influence over umbilical cord blood DHA and AA status in our study. Indeed, the human placenta does not seem to produce considerable amounts of LCPUFA for fetal supply [34,35], and, although there is evidence that the fetus is capable of synthesizing LCPUFA, especially of the *n*-6 series, it is still unclear to what extent fetal fatty acid status is determined by endogenous conversion [36].

One possible explanation for the reduced levels of AA in umbilical cord blood despite increased concentration in placenta would be an enhanced placental tissue retention and metabolism of this *n*-6 LCPUFA in adolescents. In fact, there is evidence to suggest that AA accumulation in human placenta is greater than that of DHA [35,37], and this could possibly be exacerbated in adolescent pregnancies. In our study, the tissue ratio of total *n*-6/*n*-3 was significantly increased in adolescent placentas. Furthermore, studies demonstrate that AA is not only quantitatively important as a constituent of placental membranes [38], but also plays a key role in the initiation and progression of parturition through the production of eicosanoids. In particular, prostaglandins have been widely shown to mediate uterine contractility, cervical ripening and membrane rupture [39]. In that respect, pharmacological inhibition of AA metabolites biosynthesis in uterine tissues displays tocolytic effects [40] and is used to suppress premature labor [41]. This could be related to our observation that the mean duration of gestation in adolescents, although still within normal ranges, was one week shorter than in adults, further supporting the hypothesis of increased placental AA accumulation and metabolism in this group.

On the other hand, the observation that lower amounts of DHA in maternal erythrocytes and placenta of adolescents were not followed by a significant reduction in DHA content in cord blood might be related to the preferential transfer of this LCPUFA to the fetus. In the study of Haggarty et al. [35] with human placentas perfused ex vivo, DHA displayed the highest rate of transfer from the maternal to the fetal circulation, compared to ALA, LA, and AA. Furthermore, it has been shown in human placental choriocarcinoma cells that almost 60% of the DHA is esterified into triacylglycerol pools after cellular uptake whereas only 37% is found in phospholipid fractions [37]. These observations suggest that DHA may be preferentially available for transport to the fetal circulation, rather than being incorporated into placental membranes. This increased selectivity for transfer and lower tissue retention of DHA are mechanisms that could be protecting fetal DHA status from substantial ablation despite lower maternal and placental tissue levels in the adolescent group.

In the present study, placenta from adolescent mothers displayed decreased expression of PPARγ protein content, CD36 and FABP3 mRNA as well as FATP1 mRNA and protein levels, but no difference in FABPpm mRNA and FATP4 mRNA and protein expressions. Despite the significant downregulation of three out of the five analyzed placental transporters thought to mediate fatty acid uptake, intracellular trafficking and transfer to the fetal circulation [2,11], only a mild decrease of about 10% in total LCPUFA and 12% of AA levels were detected in cord blood of the adolescent group. Moreover, even though DHA content was approximately 18% lower in maternal erythrocytes, only a non-significant decrease of around 10% was detected in umbilical cord of this group. This could suggest a protective mechanism to ensure DHA fetal supply in adolescents. In addition, although there is still insufficient evidence regarding regulation of these proteins across pregnancy, a study in rats suggests that gestational age influences the expression of some genes involved in uptake, trafficking and synthesis of FA in placenta [42]. In our study, the expression of placental FA transporters was assessed at term, however erythrocytes reflect long-term fatty acid status due to their slow turnover rates (half-life of 120 days) and low sensitivity to recent lipid intake [43]. Thus, we cannot fully discard the possibility that differences in FA transporter expression might have occurred earlier in pregnancy and influenced long-term FA status.

The concomitant downregulation of both FABP3 and CD36 found in our study is not surprising, since these proteins have been shown to interact and be co-expressed in different animal tissues [44,45], suggesting coordinated action within the cell [10]. Even though the precise role of FABP3 in the human placenta remains unclear, FABP3 has been shown to preferentially bind *n*-6 PUFA [46]. Moreover, in placentas from Fabp3-knockout mice, transport coefficients were reduced by 44% for LA and 17% for ALA, compared to wild-type mice at the end of pregnancy [12], suggesting that this FABP regulates PUFA transport.

When studying the effects of DHA supplementation during pregnancy on the expression of placental lipid carriers, Larqué et al. [9] found that FATP1 and FATP4 mRNA levels were directly correlated with DHA content in both maternal plasma and placental phospholipids. In our study, lower DHA status in maternal blood and placenta of adolescents was accompanied by decreased expression of FATP1, however FATP4 was not different at both mRNA and protein levels. Interestingly, only FATP4 expression was significantly correlated with DHA in cord blood phospholipids, suggesting involvement of this protein in the selective placental transport of *n*-3 LCPUFA [9]. Studies by Campbell et al. [8,47] demonstrated that placental FABPpm displays higher affinities and binding capacities for LCPUFA compared with other radiolabeled fatty acids, and that inhibition of this protein impairs fatty acid uptake in the order of DHA > AA > ALA > LA >> oleic acid, further indicating that placental FABPpm is involved in preferential uptake of LCPUFA, especially DHA. In line with these observations, it is possible that the normal placental expression of FATP4 and FABPpm found in our study could contribute to prevent DHA levels from being significantly altered and to keep total LCPUFA and AA only mildly decreased in cord blood erythrocytes of the adolescents from our study.

Placental FA uptake and metabolism are also partly regulated by the nuclear receptors PPARs [48]. Treatment of pregnant mice with the PPARγ agonist rosiglitazone has been shown to enhance the expression of FATP1, FATP4, CD36 and FABPpm [49]. Similar results have been demonstrated in cultured primary human trophoblasts, in which FATP1 and FATP4 mRNA levels were increased following treatment with both PPARγ and RXR agonists [50]. In our study, lower PPARγ protein expression in placenta from adolescents was accompanied by reduced levels of only FATP1 and CD36, but no difference was observed in FATP4 and FABPpm expression. It should be noted that PPARγ transcriptional regulation of target genes occurs in response to binding small lipophilic ligands such as specific FA and derivates [51], however PPARγ activity was not evaluated in the present study. On the other hand, lower PPARγ protein expression in placentas from the adolescent group could possibly be related to their decreased DHA concentration, as it has been demonstrated in Reh and Ramos cells that DHA induces PPARγ protein levels [52], although causality cannot be inferred.

## 5. Conclusions

Conclusions drawn from this study have limitations. The major limitation is that it is a pilot study with a convenience and small sample size that hampers representativeness for the general Brazilian population. The small number of individuals also made it difficult to perform statistical analyses of association and regression that could yield information about the contribution of factors to the analyzed outcomes. Despite this, it was possible to detect differences in fatty acid concentrations and expression of genes and proteins involved in their placental transport and metabolism, in the pairwise comparisons between adolescents and adults. A second limitation of this study is that other membrane bound and cytosolic fatty acid binding proteins, lipases and lipoprotein receptors also mediate the transfer of fatty acids across the placenta and were not evaluated here. In summary, our findings show that even though some of the analyzed targets involved in lipid uptake, transfer, cytosolic trafficking and metabolism were downregulated in the placenta of adolescents, no difference was found in part of the analyzed targets and fetal FA status seemed to be mostly preserved. Total LCPUFA and AA levels were only moderately lower in cord blood and, in particular, no significant difference was observed in DHA content despite lower maternal status of this *n*-3 LCPUFA in the adolescent group. In addition, no alterations in the analyzed neonatal parameters were detected indicating that, overall, metabolic and molecular adaptations might have occurred in adolescent pregnancies from our study in order to protect fetal health.

## Figures and Tables

**Table 1:** List of primers used for quantitative RT-PCR.

Gene	Forward Primer (5′–3′)	Reverse Primer (5′–3′)
FAT/CD36	GGAAAGTCACTGCGACATGA	CCTTGGATGGAAGAACGAATC
FABPpm	GGAAGGAAATAGCAACAGTGG	TCCTACACGCTCACCATATAAGC
FATP1	AGGTGGTTCAGTACATCGGG	AGAACTCCCCGATTTGGC
FATP4	ATACCCACTGAACCTTTGGC	AAGGTCTCTGTGGTGGCCAA
FABP3	TTTTGCTACCAGGCAGGTG	TCATCTGCTGTTGTCTCATCG
GAPDH	GAAGGTGAAGGTCGGAGTCAA	GGAAGATGGTGATGGGATTTC

**Table 2:** General characteristics of the adult and adolescent mothers.

Variables	Adult (*n* = 15)	Adolescent (*n* = 15)
**Maternal age (year) (mean ± SD (range))**	25.6 ± 5.5 (20–35)	16.9 ± 1.1 (15–19)
**Per capita household income (n (%))**		
<1 minimum wage	10 (66.7)	10 (66.7)
≥1 minimum wage	3 (20)	2 (13.3)
Unable to inform	2 (13.3)	3 (20)
**Marital status (n (%))**		
Single	5 (33.3)	12 (80)
Married or co-habiting	10 (66.7)	3 (20)
**Education (n (%))**		
<8 years	4 (26.6)	9 (60)
≥8 years	11 (73.3)	6 (40)
**Employment during pregnancy (n (%))**		
Yes	12 (80)	1 (6.7)
No	3 (20)	14 (93.3)
**Parity (n (%))**		
No delivery	8 (53.3)	13 (86.7)
≥1 delivery	7 (46.7)	2 (13.3)
**Prenatal follow-up visits (n (%))**		
<6	1 (7)	2 (13.3)
≥6	14 (93)	13 (86.7)

**Table 3:** Anthropometric variables and obstetric outcomes of adult and adolescent pregnancies.

Variables	Adult	Adolescent
***Maternal Characteristics***		
**Pre-pregnancy nutritional status (n (%)) ^a^**		
Underweight	0	0
Normal weight	10 (66.7)	11 (78.6)
Overweight	5 (33.3)	4 (21.4)
**Gestational weight gain (n (%)) ^a^**		
Insufficient	1 (6.7)	4 (28.0)
Recommended	8 (53.3)	6 (36.0)
Excessive	6 (40.0)	5 (36.0)
**Delivery mode (n (%))**		
Vaginal	11 (73.3)	11 (73.3)
Cesarean section	4 (26.7)	4 (26.7)
***Neonatal characteristics***		
**Newborn sex (n (%))**		
Females	7 (46.7)	10 (66.7)
Males	8 (53.3)	5 (33.3)
**Birth weight (g) (means ± SD)**	3347 ± 350	3395 ± 346
**Length at birth (cm) (means ± SD)**	47.4 ± 5.9	48.6 ± 1.5
**Head circumference (cm) (means ± SD)**	34.6 ± 3.7	34.3 ± 1.6
**Placental weight (g) (means ± SD)**	661 ± 130	610 ± 103
**Gestational age at delivery (weeks)**	39.9 ± 0.8	38.9 ± 0.7 *
**Birth weight classification (n (%)) ^b^**		
SGA	0	0
AGA	15 (100)	15 (100)
LGA	0	0

^a^ According to IOM. Weight Gain during Pregnancy: Reexamining the Guidelines. Institute of Medicine (US) and National Research Council (US) and Committee to Reexamine IOM Pregnancy Weight Guidelines; 2009. ^b^ According to Villar J., Cheikh Ismail L., Victora C. G., et al. International standards for newborn weight, length, and head circumference by gestational age and sex: the Newborn Cross-Sectional Study of the INTERGROWTH-21st Project. Lancet 2014; 384:857-868. LGA, large-for-gestational-age; SGA, small-for-gestational-age; AGA, appropriate-for-gestational-age; * *p* < 0.05, Adolescents (*n* = 15) vs. Adults (*n* = 15) according to Mann-Whitney’s test.

**Table 4:** Placental fatty acid concentrations in adult and adolescent pregnancies.

	Placenta		
Fatty acid (mg/100mg fatty acids)	Adult	Adolescent	*p*
**Saturated (SFA)**			
16:0	25.0 ± 3.5	23.0 ± 1.1	0.06
18:0	13.6 ± 1.2	13.3 ± 1.0	0.38
**Monounsaturated (MUFA)**			
18:1 *n*-9 *cis*	**8.5 ± 1.5**	**11.0 ± 1.6**	**0.01**
18:1 trans *	0.95 ± 0.8	1.1 ± 0.6	0.39
**Essential polyunsaturated (EFA)**			
18:2 *n*-6 (LA)	**8.4 ± 1.5**	**9.6 ± 1.0**	**0.01**
18:3 *n*-3 (ALA)	0.80 ± 0.4	0.92 ± 0.3	0.41
**Long-chain polyunsaturated (LCPUFA)**			
20:4 *n*-6 (AA)	**14.6 ± 4.5**	**17.2 ± 1.6**	**0.04**
22:4 *n*-6	0.98 ± 0.2	0.90 ± 0.3	0.94
20:5 *n*-3 (EPA)	0.44 ± 0.2	0.29 ± 0.1	0.35
22:6 *n*-3 (DHA)	**2.4 ± 0.6**	**1.3 ± 0.9**	**0.001**
22:5 *n*-3 (DPA)	0.87 ± 0.5	0.56 ± 0.4	0.23
**Total SFA ^a^**	44.5 ± 7.0	42.1 ± 2.4	0.21
**Total MUFA ^b^**	**18.8 ± 4.0**	**25.1 ± 4.4**	**0.04**
**Total EFA ^c^**	**9.2 ± 1.7**	**10.6 ± 1.1**	**0.01**
**Total LCPUFA ^d^**	22.0 ± 6.2	22.8 ± 5.4	0.73
**Total PUFA *n*-6 ^e^**	**26.7 ± 1.9**	**31.3 ± 5.4**	**0.04**
**Total PUFA *n*-3 ^f^**	**5.8 ± 0.4**	**4.3 ± 1.1**	**0.01**
**Total *n*-6/*n*-3 ratio**	**5.0 ± 1.3**	**7.7 ± 2.4**	**0.0007**
**AA/LA *n*-6 ratio**	1.75 ± 0.6	1.78 ± 0.1	0.88
**DHA/ALA *n*-3 ratio**	**4.8 ± 2.8**	**1.5 ± 1.0**	**0.01**

Values given are mean ± standard deviation (SD). AA—arachidonic acid; EPA—eicosapentaenoic acid; DHA—docosahexaenoic acid; DPA—docosapentaenoic acid; ^a^ total SFA includes 14:0, 16:0, 18:0 and 22:0; ^b^ total MUFA includes 18:1 9c and 18:1 11c; ^c^ total EFA includes 18:3 *n*-3 and 18:2 *n*-6; ^d^ total LCPUFA includes 20:3 *n*-6, AA, EPA,22:4 *n*-6, 22:5 *n*-3 and DHA; ^e^ total PUFA *n*-6 includes 18:2 *n*-6, 18:3 *n*-6, 20:3 *n*-6, 20:4 *n*-6 and 22:4 *n*-6; ^f^ total PUFA *n*-3 includes 18:3 *n*-3, 20:5 *n*-3, 22:5 *n*-3 and 22:6 *n*-3. * Includes 18:1 *n*-9 *trans* and 18:1 *n*-11 *trans*. Results and *p* values in bold denote significant difference according to unpaired *t* test, comparing Adolescents (*n* = 15) vs. Adults (*n* = 15).

**Table 5:** Fatty acid composition of maternal and cord blood erythrocytes from adults and adolescents.

	Maternal Erythrocytes		Umbilical Cord Erythrocytes	
Fatty Acids (mg/100 mg)	Adult	Adolescent	Adult	Adolescent
**Saturated (SFA)**				
14:0	0.5 ± 0.1	0.5 ± 0.1	0.5 ± 0.1	0.7 ± 0.2 **
16:0	23.5 ±1.4	23.6 ± 1.2	23.9 ± 0.9	25.8 ± 2.4 *^,^#
18:0	17.4 ± 0.8	17.7 ± 1.2	18.5 ± 0.5 ***	19.4 ± 1.8 **
22:0	0.8 ± 0.13	0.7 ± 0.20	0.7 ± 0.1	0.8 ± 0.45
**Monounsaturated (MUFA)**				
18:1 *n*-9 *cis*	12.5 ± 1.3	11.9 ± 1.0	10.9 ± 1.2 ***	10.4 ± 1.7 ***
18:1 *n*-7 *cis*	1.2 ± 0.2	1.3 ± 0.3	1.4 ± 0.2	1.5 ± 0.3
**Essential polyunsaturated (EFA)**				
18:2 *n*-6 (LA)	11.0 ± 1.8	12.0 ± 1.0	4.2 ± 0.6 ***	4.3 ± 0.7 ***
18:3 *n*-3 (ALA)	0.3 ± 0.1	0.4 ± 0.2	0.2 ± 0.1	0.3 ± 0.1
**Long-chain polyunsaturated (LCPUFA)**			
20:4 *n*-6 (AA)	15.8 ± 1.6	14.7 ± 1.4	20.0 ± 1.6 ***	17.6 ± 2.5 ***^,^#
22:4 *n*-6	3.8 ± 0.6	4.5 ± 0.7 #	4.3 ± 0.4	4.1 ± 1.0 *
20:5 *n*-3 (EPA)	0.7 ± 0.3	0.5 ± 0.3	0.8 ± 0.1	0.8 ± 0.2 *
22:6 *n*-3 (DHA)	5.5 ± 1.3	4.5 ± 0.5 #	6.1 ± 0.9	5.5 ± 1.0 **
22:5 *n*-3 (DPA)	1.9 ± 0.5	1.8 ± 0.3	0.5 ± 0.2 ***	0.6 ± 0.2 ***
**Total SFA** ^a^	41.8 ± 2.7	42.3 ± 1.9	43.7 ± 1.0*	47.5 ± 4.4 **
**Total MUFA** ^b^	13.8 ±1.4	13.1 ± 0.9	12.3 ± 1.3	12.0 ± 1.8
**Total EFA** ^c^	11.5 ± 1.8	12.5 ± 1.0	4.5 ± 0.6 ***	4.6 ± 0.6 ***
**Total LCPUFA** ^d^	30.3 ± 3.2	28.0 ± 2.3 #	34.9 ± 1.8 ***	31.5 ± 3.5 ***^,^##
**Total PUFA *n*-6** ^e^	33.5 ± 2.4	33.8 ± 2.3	32.0 ± 1.8	30.0 ± 1.4 *
**Total PUFA *n*-3** ^f^	8.5 ±1.2	7.2 ± 0.6 ##	7.7 ± 1.1	7.2 ± 1.0
**Total *n*-6/*n*-3 ratio**	4.1 ± 0.8	4.7 ± 0.5 #	4.3 ± 0.8	4.1 ± 0.7 *
**AA/LA *n*-6 ratio**	1.5 ± 0.7	1.3 ± 0.2	4.8 ± 0.5	4.2 ± 0.7
**DHA/ALA *n*-3 ratio**	18.4 ± 1.6	11.4 ± 1.5 #	30.5 ± 2.7 ***	18.3 ± 1.7 ***^,^#

Values are expressed as mean ± standard deviation (SD). LA, linoleic acid; ALA, alpha-linolenic acid; AA, arachidonic acid; EPA, eicosapentaenoic acid; DHA, docosahexaenoic acid; DPA, docosapentaenoic acid; ^a^ total SFA includes 14:0, 16:0, 18:0 and 22:0; ^b^ total MUFA includes 18:1 9c and 18:1 7c; ^c^ total EFA includes 18:3 *n*-3 and 18:2 *n*-6; ^d^ total LCPUFA includes 20:3 *n*-6, AA, EPA,22:4 *n*-6, 22:5 *n*-3 and DHA; ^e^ total PUFA *n*-6 includes 18:2 *n*-6, 18:3 *n*-6, 20:3 *n*-6, 20:4 *n*-6 and 22:4 *n*-6; ^f^ total PUFA *n*-3 includes 18:3 *n*-3, 20:5 *n*-3, 22:5 *n*-3 and 22:6 *n*-3. Statistical difference between maternal and umbilical cord erythrocytes within each group is denoted by * *p* < 0.05, ** *p* < 0.01 and *** *p* < 0.001, according to paired *t* test. Statistical difference in maternal or umbilical cord erythrocytes between groups is denoted by # *p* < 0.05 and ## *p* < 0.01, according to unpaired *t* test.

## Supplementary Materials

The following is available online at http://www.mdpi.com/2072-6643/10/2/220/s1, Figure S1: Placental protein expression of FATP-1 and FATP-4.
