# Supplementary material for: Differential Long-Chain Polyunsaturated Fatty Acids Status and Placental Transport in Adolescent Pregnancies
Source: Nutrients. 2018 Feb 15;10(2):220. doi: 10.3390/nu10020220 (PMC5852796; doi:10.3390/nu10020220)
Supplement: Supplementary file 1 [file nutrients-10-00220-s001.pdf]

nutrients

MDPI

Supplementary Materials:

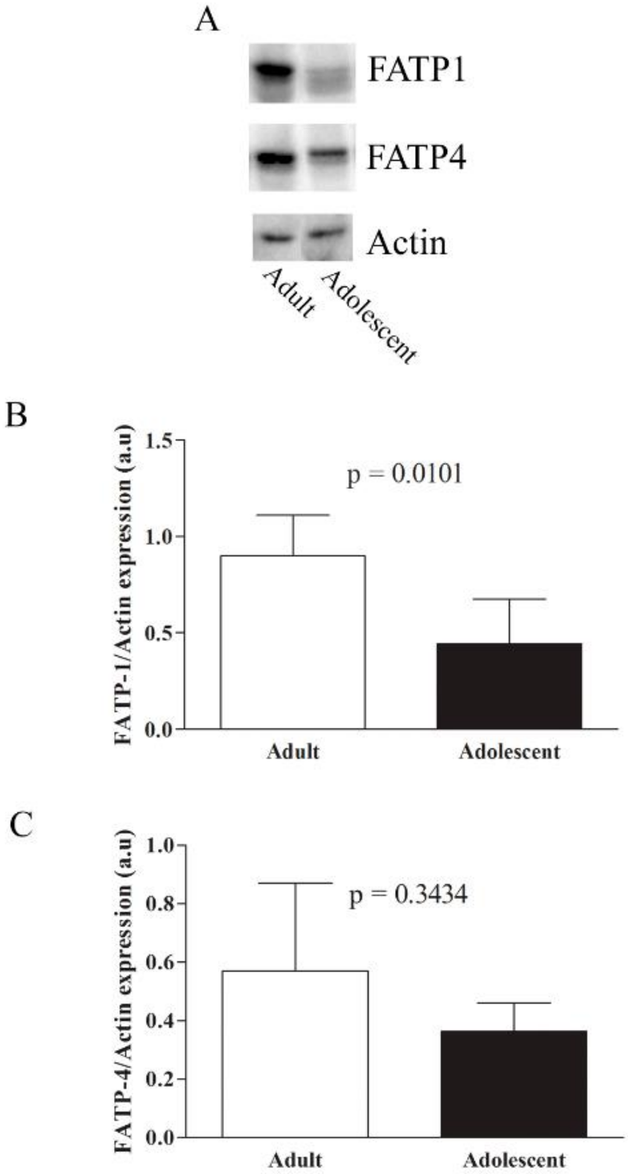

Figure S1. Placental protein expression of FATP1 and FATP4. (**A**) Placental protein expression of FATP1 and FATP4 in adults (n = 7) and adolescents (n = 6). Placental densitometric analysis of (**B**) FATP1 and (**C**) FATP4 after normalization by actin. Results are expressed as mean ± SD. $p < 0.05$ was considered significantly different according to unpaired t test.

© 2018 by the authors. Submitted for possible open access publication under the terms and conditions of the Creative Commons Attribution (CC BY) license (http://creativecommons.org/licenses/by/4.0/).
